# An Improved Dyna-Q Algorithm Inspired by the Forward Prediction Mechanism in the Rat Brain for Mobile Robot Path Planning

**DOI:** 10.3390/biomimetics9060315

**Published:** 2024-05-23

**Authors:** Jing Huang, Ziheng Zhang, Xiaogang Ruan

**Affiliations:** 1 Faculty of Information Technology, Beijing University of Technology, Beijing 100124, China; 2 Beijing Key Laboratory of Computational Intelligence and Intelligent System, Beijing 100124, China

**Keywords:** VTE, hippocampus forward prediction, Dyna-Q, mobile robots, path planning, brain-inspired computing

## Abstract

The traditional Model-Based Reinforcement Learning (MBRL) algorithm has high computational cost, poor convergence, and poor performance in robot spatial cognition and navigation tasks, and it cannot fully explain the ability of animals to quickly adapt to environmental changes and learn a variety of complex tasks. Studies have shown that vicarious trial and error (VTE) and the hippocampus forward prediction mechanism in rats and other mammals can be used as key components of action selection in MBRL to support “goal-oriented” behavior. Therefore, we propose an improved Dyna-Q algorithm inspired by the forward prediction mechanism of the hippocampus to solve the above problems and tackle the exploration–exploitation dilemma of Reinforcement Learning (RL). This algorithm alternately presents the potential path in the future for mobile robots and dynamically adjusts the sweep length according to the decision certainty, so as to determine action selection. We test the performance of the algorithm in a two-dimensional maze environment with static and dynamic obstacles, respectively. Compared with classic RL algorithms like State-Action-Reward-State-Action (SARSA) and Dyna-Q, the algorithm can speed up spatial cognition and improve the global search ability of path planning. In addition, our method reflects key features of how the brain organizes MBRL to effectively solve difficult tasks such as navigation, and it provides a new idea for spatial cognitive tasks from a biological perspective.

## 1. Introduction

As a hot field, robots have been widely concerned. In recent years, a lot of research has been carried out around robots’ environmental cognition, path planning, mobile obstacle avoidance, and other tasks. Among them, spatial cognition and path planning are necessary functions of mobile robots [1]. The path planning of mobile robots mainly includes two types:

(1) Global path planning based on complete environmental prior information. Under the condition that the external environment is known, the robot can use traditional global path planning algorithms such as A* [2] or Dijkstra algorithms to generate the best path in the existing environmental map. However, known environmental information is not always complete and accurate, and there may be deviations or unknowns in some areas.
(2) Local path planning with uncertain environmental information, such as the dynamic window method (DWA) [3]. In the absence of environmental knowledge, such methods may fall into local optimization. They only simulate and evaluate the next action and are prone to getting into trouble when encountering ‘C’ obstacles.

The two kinds of methods both rely on the environmental map for path planning, lack the ability to learn autonomously, and are unable to complete autonomous space cognition. Moreover, users need to pre-program every situation they may encounter, which will bring high costs.

Therefore, in order for mobile robots to explore and navigate independently in unknown environments, a learning-based approach is needed. Reinforcement Learning (RL) [4] is considered an important method for achieving general artificial intelligence by interacting with the environment in a trial-and-error manner. Since RL can learn actively and adapt to a complex dynamic environment, it provides a new method for solving the problem of path planning. Researchers have introduced RL into path planning [5,6] to address the limitations of traditional methods.

Reinforcement Learning can be divided into Model-Free Reinforcement Learning (MFRL) and Model-Based Reinforcement Learning (MBRL) according to whether there is an environment model in the algorithm. In MFRL, the agent uses the experience gained through direct interaction with the environment to improve the strategy or estimate the value function. Classic MFRL methods include Q-learning and State-Action-Reward-State-Action (SARSA) algorithms. The Q-learning algorithm and its variants are widely used in robot path planning because they can learn independently without an environmental map [7,8,9,10]. However, as the scale of the environment expands and complexity increases, such model-free methods have the disadvantages of low exploration efficiency and slow convergence. Because these methods require huge amounts of interaction with the real environment, they will generate many invalid experiences, making robots easily hit walls or causing other accidents.

By contrast, MBRL includes not only direct interaction with the environment but also a virtual environment model. The agent uses the experience gained from exploring the real environment to build a virtual environment model and obtain strategies, and the agent uses this strategy to continue to collect experience and expand the model in the real environment. Using the virtual environment model to make decisions can reduce the number of trials and errors in the real environment and obtain good performance strategies, effectively reduce the probability of accidents in the robot exploration process, and enhance navigation efficiency. At the same time, the new planning process can make full use of the experience gained from interacting with the real environment and improve sample utilization.

Dyna [11] is a classic MBRL framework, which includes strategic learning and internal model building. Dyna-Q is the application of the Dyna framework in Q-learning. Some researchers [12,13] have applied the Dyna-Q algorithm to robot navigation, which works well under the condition that the obstacle is static but not well in the dynamic environment. First of all, the sparsity of environmental rewards makes it difficult for robots to find the target point and the learning efficiency is low. Secondly, it is still a challenge to plan an efficient and collision-free path in the environment with multiple dynamic obstacles [14]. In addition, RL algorithms, including Dyna-Q, are generally faced with the dilemma of exploration and exploitation. Robots need to constantly try new actions to prevent falling into the local optimum and gradually converge to the optimal strategy. How to balance exploration and exploitation remains a major challenge. In general, RL has the ability for autonomous learning compared to traditional path planning algorithms, but there are still shortcomings in learning efficiency, especially in dynamic environments.

Realizing spatial cognition in a strange environment is an important survival skill for mammals. Many studies have shown that spatial cognitive function plays an important role in animal navigation [15]. Studying the spatial cognitive mechanism of animals is of great significance for people to improve existing navigation methods and to imitate and learn from unique mechanisms in biology. Animals have excellent navigation skills. Tolman [16] found that mice could explore and learn the structure of a maze independently. Inspired by the spatial cognition and navigation functions of animals, researchers use the neurophysiological mechanism in animals for reference to conduct computational modeling, further deepening the understanding of the cognitive mechanism of animals and providing new ideas for the navigation of mobile robots.

Following such a research approach, this paper presents an improved Dyna-Q algorithm inspired by cognitive mechanisms in the hippocampus and striatum to achieve more efficient navigation in unknown environments or without sufficient environmental knowledge.

The main contributions of this paper are as follows:

(1) We propose an improved Dyna-Q algorithm inspired by the cognitive mechanisms in the hippocampus and striatum. This model combines the forward prediction mechanism of the hippocampus to improve Dyna-Q’s action decision-making mechanism, so that the robot can virtually simulate the future multi-step operation when making action selection. This forward prediction mechanism can balance exploration and exploitation and reduce the probability of falling into the local optimum in navigation tasks. At the same time, it simulates the striatum function, evaluates the decision certainty of each forward simulation, and dynamically adjusts the sweep depth and action selection mode to improve the convergence and decision-making efficiency of the algorithm.
(2) The experiment carried out in the T-maze in this paper verified that this model can show characteristics similar to the VTE mechanism of rats, proved that this model has biological rationality, demonstrated the feasibility of introducing the biological neural mechanism into the machine learning method, and provided a new idea for improving the existing RL algorithm and the robot’s path planning task from a brain-like perspective.
(3) This paper implements robot navigation in unknown environments with static and dynamic obstacles and compares it with existing RL algorithms. Experimental results show that our algorithm achieves autonomous spatial cognition, can converge faster, and has better performance in path planning compared to the SARSA and Dyna-Q algorithms.
(4) In summary, we propose a novel Dyna-Q algorithm framework simulating cognitive mechanisms in the hippocampus and striatum to improve the efficiency of navigation tasks in complex environments, providing a promising direction for future research on RL.

The rest of this paper is as follows: Section 2 mainly introduces the relevant research background. Section 3 introduces the framework, mathematical model, and working principle of the algorithm. Section 4 presents the experimental design and results. We carry out simulation experiments in a two-dimensional maze to test the model’s spatial cognition ability, and we compare our model with other models in terms of navigation performance. In Section 5, we discuss and analyze the experimental results and possible reasons for our findings. Finally, we summarize this work and draw conclusions in Section 6.

## 2. Related Works

### 2.1. Vicarious Trial and Error

Learning to predict long-term rewards is the foundation for the survival of many animals, which is actually the goal of RL. There is evidence that brain evolution has taken many ways [17] to achieve this goal. One is the learning environment model or cognitive map, which simulates the future state to generate predictions of long-term rewards. Tolman and other researchers have noticed that when rats explore a maze and encounter choice points such as crossroads, they occasionally stop and wander back and forth, which seems to indicate confusing about which path to take. These researchers speculated that rats are imagining potential future choices and called this behavior Vicarious Trial and Error (VTE) [18].

**Figure 1:**
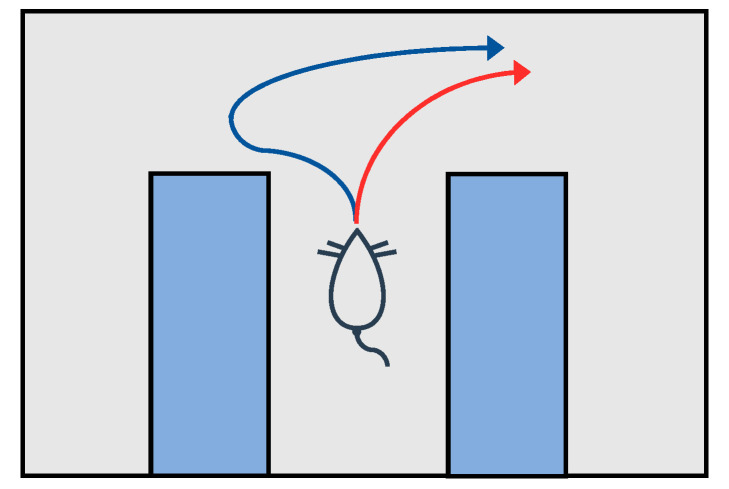
Vicarious Trial and Error [18]. In Tolman’s view, VTE is a prospective imagination of the future and fundamentally a behavioral observation of pause and reorientation. VTE reflects forward imagination and an assessment of the future. The blue line shows that rats pause and deliberate when they find it difficult to choose a point. The red line is the behavior without the VTE mechanism. The rats select only one track at the selection point and continue along that track.

When making decisions, biological agents have a process of deliberation [19], which is based on a schema describing how the world works, such as a cognitive map, to evaluate potential possibilities, and they use the results of these assumptions as a means of decision making. VTE usually occurs in the early stages of rat learning [20], especially when rats do not know what action to take in certain positions after random exploration and a preliminary understanding of space. The VTE process is shown in Figure 1.

Current research on the mechanism of VTE is mainly focused on its biological function and explanation. Researchers have placed rats in an environment with a fork in the road, such as a T-maze, to test rat behavior characteristics during VTE. VTE is considered to reflect biological imagination and an assessment [21] of the future, so it is a flexible and deliberative decision-making process.

**Figure 2:**
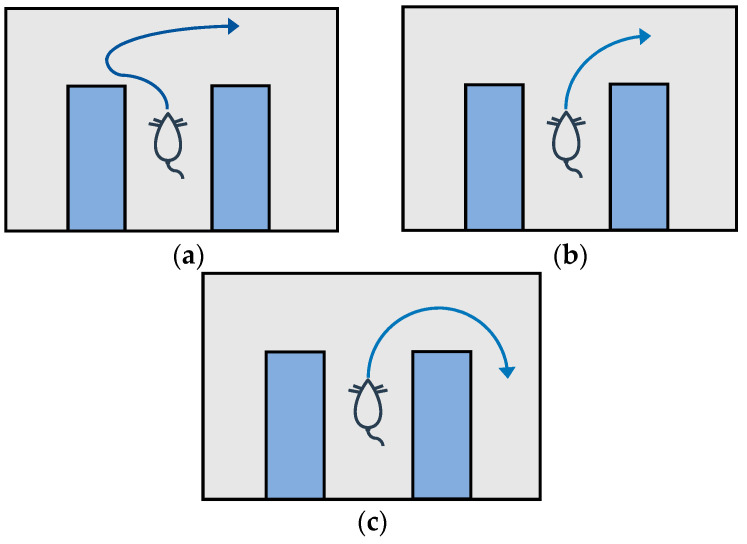
Three stages of the VTE process [18]. These are deliberation, planning, and automation, respectively. (**a**) In the first stage, rats have a preliminary understanding of the structure of the environment, but need to imagine different schemes indirectly to make a final decision. (**b**) In the second stage, rats are familiar with the structure of the environment and have a relatively definite behavior plan, but they are still in a deliberate state. They just keep exploring one option at a time to make sure it is the option they want. (**c**) In the third stage, automation, rats will no longer virtually search for possible tracks but will confidently execute a certain sequence of actions.

The VTE process is usually divided into three stages: deliberation (the VTE process is significantly enhanced), planning (the VTE process is gradually reduced), and automation (VTE is no longer performed, but a certain sequence of actions is performed), as shown in Figure 2. Studies have shown that in goal-oriented navigation, the VTE mechanism is related to the hippocampus-ventral striatum circuit [22] in the rodent brain. When navigating the maze, rats will stop at the decision point, first turning their head in one possible direction, and then turning it in another. In the process of turning back and forth, the place cells corresponding to the selected branch of the maze are activated after forward sweeping, like the rats were really passing through that path.

The VTE mechanism allows rats and other mammals to simulate the possible trajectory and locations in the brain in the next few steps when they face multiple paths from which to choose, such as a fork. Therefore, animals can evaluate the effect of different paths, thus improving the efficiency of spatial cognition and path planning.

As the VTE mechanism is beneficial for spatial cognition and goal-directed learning of animals, imitating it and presenting a novel algorithm would help increase the efficiency of robot navigation and improve RL algorithms by optimizing decision-making policies, which is the motivation for our work.

### 2.2. Forward Prediction and Decision Certainty Assessment

Adaptive behavior of animals in the environment requires the ability to analyze past experience, which is often forward looking and retrospective, and hippocampal function is crucial for the representation and storage of sequence information. Forward prediction represented by the hippocampal theta oscillation [23] is considered an important part of the VTE process. Rats will carry out “mental travel” to simulate the possible results. Place cells [24] in the CA1 region of the rat hippocampus can encode spatial information and activate at a specific location. Their spatial specificity allows rats to position themselves in space. In the active navigation process, place cells are internally organized, generating forward and reverse sequences in a single period of theta oscillation.

Theta sequences in the hippocampus may be the basis for human situational memory retrieval [25], which can ensure the accuracy and stability of spatial representation of place cells. Moreover, goal-directed navigation is difficult to support [26] without theta sequences. Therefore, the forward sweep mechanism based on theta sequences is crucial for memory-guided behavior in the hippocampus [27].

**Figure 3:**
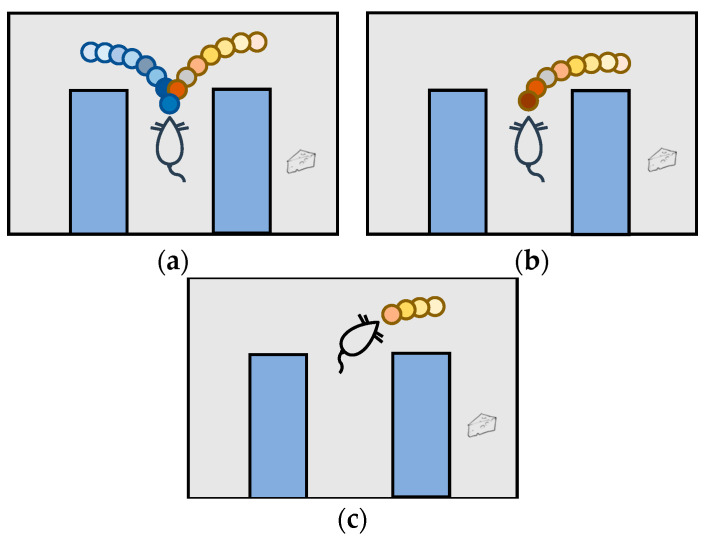
Forward prediction of the hippocampus [18]. Researchers trained rats to find a way to obtain rewards (cheese) in the environment with a fork in the road. (**a**) In the deliberation phase of VTE, sweeps are conducted in different directions to simulate as many paths as possible. In the early stages of training, due to a lack of confidence in decision making, place cells in the hippocampus of rats were activated in turn, simulating possible future spatial trajectories. The blue and yellow tracks represent activation sequences in different directions in the hippocampus. (**b**) In the second stage, the rat’s decision certainty is increasing and the sweep effect of the rat hippocampus is also gradually weakened, and the sweep tends to move toward the goal. (**c**) In the third stage, rat behavior tends to develop toward automation and tends to advance along a fixed sequence of actions. The length of the hippocampus sweep is decreased, mainly in the determined direction.

Theta oscillation occurs when rats stop at the selection point and exhibit VTE. In a given theta cycle, place cells activate sequentially along a virtual path and move toward a goal [28], then the next cycle begins to forward sweep in another direction. The place cell activation sequence alternates between possible future paths and the rat’s moving direction. In this way, animals virtually attempt possible future actions and observe potential outcomes of these actions in the brain, ultimately forming multiple potential pathways. The hippocampus forward prediction sequence is shown in Figure 3.

Neurophysiological studies show that the forward sweep mechanism of place cells is not a way to collect external environmental information [29]. It merely represents an alternative search process within animals, and the collection of environmental information still depends on exploration at the early stage of training. Therefore, VTE will not occur at initial exploration but only after animals have had experience with tasks and built an environment model.

On the basis of the simulation of the future path, it is also necessary to finally evaluate an optimal solution for each path, so as to improve the certainty of the decision. The striatum is adjacent to and closely connected to the hippocampus in the brain area. Research shows that the striatum is closely related to reward learning and action selection [30], and its role in animal environmental cognition is mainly to make action selection and evaluate the reward value that can be obtained by the action taken, showing a relative preference for action selection and reward expectation. Some striatum-based computational models are mainly applied to RL and action selection.

Strong projection from the CA1 region of the hippocampus to the ventral striatum (vStr) may transmit spatial context information [31,32], forming the connection between position and reward [33]. Reward-related cells in the vStr are activated during VTE to provide reward signals [34]. It receives dopamine released by dopamine cells in the ventral tegmental area (VTA) to assess the certainty of current predictions. When the hippocampus generates forward prediction sequences at difficult decision points to simulate future spatial trajectories, the ventral striatum assesses these predictions. The joint action of the two allows the animal to make a long-term plan for its own actions in mind. The above approach is similar to MBRL, which can summarize many different values, rather than representing the world from a single value level.

Existing research shows that mammalian brains can implement model-based mechanisms, that is, establish a virtual environment model inside the brain based on direct interaction with the environment, and then learn based on this model. For example, Khamassi et al. [35] reviewed model-based (preserving the representation of the world) and model-free (responding to immediate stimuli) learning algorithms, using the dorsolateral striatum to represent “model-free” learning and the dorsomedial striatum to represent “model-based” learning, and then proposed that the core role of the ventral striatum is to learn the probability of action selection of each state transition. They proposed a model-based bidirectional search model, which combines forward trajectory sampling from the current position and backward sampling by prioritized sweeping from the state related to the large reward prediction error to explain why hippocampal reactivations drastically diminish when the animal’s performance is stable. Elisa Massi et al. [36] imitated hippocampal activations, implemented an experience replay mechanism, and applied it to mobile robot navigation, giving the navigation robot a neuro-inspired RL architecture.

Stoianov et al. [37] proposed a spatial navigation calculation model based on the hippocampus-ventral striatum (HC-vStr) circuit, proving the possibility of mapping and simulating the MBRL mechanism with the HC-vStr circuit to reproduce behavior and neural data. They used a Bayesian nonparametric model to build a brain-inspired MBRL calculation model, and verified that this forward looking prediction in the rat brain could improve action selection and learning. Chai et al. [38] proposed a striatal behavior learning model consisting of striatum and matrix model to explain the generation of habitual behavior in animal navigation. In the striatum model, directional information is constantly updated based on the mechanism of operant conditioning, which leads to habitual behavior. They tested the Morris square dry maze task, and the results showed that the model was effective in explaining habit-related behavior. It could successfully solve navigation tasks with habits and display key neural characteristics of the striatum, which may be significant for the bionic navigation of robots.

As can be seen from the above studies, the introduction of the above-mentioned forward prediction and decision certainty assessment mechanism into an RL algorithm will help to improve the robot’s environmental cognitive efficiency, reduce exploration randomness, and make full use of the knowledge obtained in the previous exploration, which is also the overall idea of this model.

### 2.3. Dyna-Q Algorithm

Q-learning is a classical MFRL algorithm for solving MDP. Its main advantage is to use the temporal difference algorithm (TD) to achieve off-policy learning. $Q_{\pi }\left(s,a\right)$ is defined by the expected return of the state-action pair $\left(s,a\right)$ under a policy. The calculation formula is as follows:

(1) $$Q_{\pi }\left(s,a\right)=E_{\pi }[G_{t}\;|\;s_{t}=s,a_{t}=a]=E_{\pi }[\;\sum_{k=0}^{\infty }\gamma^{k}r_{t+k+1}\;|\;s_{t}=s,a_{t}=a]$$

The core idea of the Q-learning algorithm is that the next Q value of the current state-action pair $\left(s,a\right)$ is generated by the strategy to be evaluated, rather than the Q value of the next state-action pair $\left(s^{\prime },a^{\prime }\right)$ following the current strategy. The final strategy of Q-learning is obtained by iterating the state-action value function $Q\left(s,a\right)$. The Q table is a set of $Q\left(s^{\prime },a^{\prime }\right)$, which is used to store the agent’s preferences for taking different actions in different states of the environment, thus promoting action selection. The update of the state-action value is regulated as follows:

(2) $$Q\left(s^{\prime },a^{\prime }\right)=Q\left(s,a\right)+\alpha \cdot [r_{t+1}+\gamma \cdot \;{max}_{a}Q\left(s^{\prime },a^{\prime }\right)-Q\left(s,a\right)]$$

**Figure 4:**
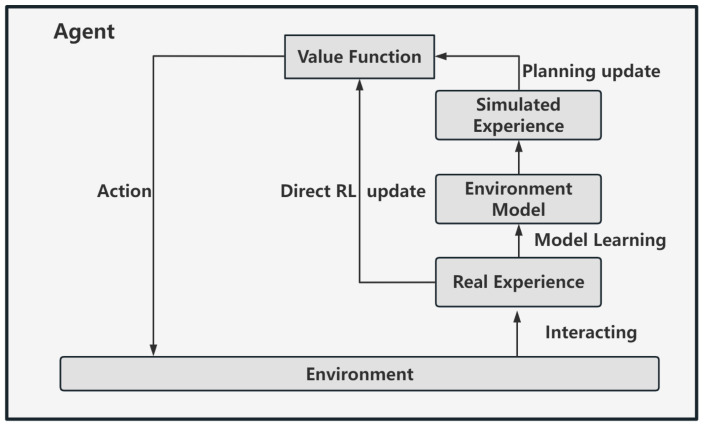
Dyna architecture. This architecture combines model-free methods with a virtual world model.

Dyna architecture is characterized by combining model-free methods with a world model, as shown in Figure 4. The world model can provide a large amount of simulation data for strategy learning of model-free algorithms. The agent interacts with the environment to obtain real data and learns the internal virtual world model. Then, the world model is used to obtain simulated interaction data between the agent and the environment based on the prediction imagination in each state to learn the value function. Although there is a certain deviation between the model and the real environment, the simulated data still have high reliability to serve as training data for RL algorithms. This method can well supplement the data needed for strategy training in model-free methods, reduce interaction and cost with the real environment, and improve sample efficiency.

Dyna-Q is the application of Dyna architecture to Q-learning. Based on Q-learning, the planning link is added to store transfer action pairs $\left(s_{t},a_{t},r_{t+1},s_{t+1}\right)$ obtained from interaction with the environment in the model. Then, data are randomly extracted from the model and planned in the Q-learning method to speed up learning efficiency.

However, Dyna-Q also has some shortcomings, as mentioned in the introduction:

(1) In the path planning problem, the reward function of traditional RL rewards the agent only when it reaches the destination or encounters obstacles. When the environment is large, there are many invalid states, and the traditional reward function has the problem of sparse reward. Therefore, it is difficult for the agent to obtain positive rewards and find the goal point. It requires a lot of random searches to gain effective experience, and learning efficiency is low.
(2) Secondly, it is still a challenge to plan an efficient and collision-free path in the environment with multiple dynamic obstacles. To achieve this goal, RL algorithms also need to increase the time cost of learning.
(3) In addition, RL algorithms, including Dyna-Q, are generally faced with the dilemma of exploration and exploitation. In the early stages of training, exploration of the agent causes severe blindness. The agent needs to constantly try new actions to prevent falling into the local optimum, reduce exploratory behavior at a later stage, and gradually converge to the optimal strategy. How to balance exploration and exploitation remains a major challenge.

In order to solve this problem, the work to be carried out in this paper introduces the mechanism in the above biological agent on the basis of Dyna-Q. This will allow the agent to make decisions no longer limited to the Q value of the current operation, but to simulate future multi-step operations before making decisions to achieve the effect of long-term planning, so that the agent can try different future tracks as much as possible at the initial stage of exploration and more effectively collect experience. After the certainty of decision making reaches a high level, the length and frequency of sweeping are gradually reduced to speed up navigation, better achieve balance between exploration and exploitation, improve the convergence property and decision-making efficiency of the agent, and finally improve the performance of traditional RL algorithms in the task of robot environmental cognition and path planning from a biological point of view.

## 3. Materials and Methods

### 3.1. Overall Framework of the Model

**Figure 5:**
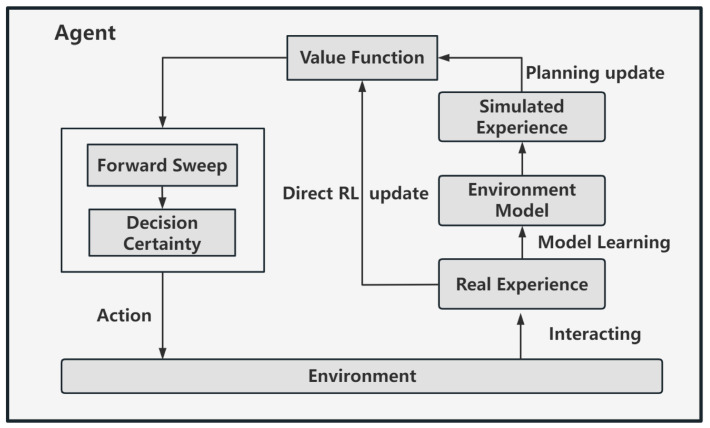
The overall framework of the model. Unlike the traditional RL algorithm, which directly selects the action of a specific state based on the Q table, this model adds forward sweep and decision certainty assessment.

Inspired by the VTE behavior of rats and other mammals in environmental cognition, we simulate the functions of brain regions such as the hippocampus and ventral striatum and introduce them into the Dyna-Q algorithm, and we propose a brain-inspired environmental cognitive computing model. The overall framework of the model is shown in Figure 5 below.

The overall framework to the right of the figure is consistent with Dyna-Q. Direct RL is based on real-world experience gained through direct interaction with the environment, and the value function is planned and updated through the virtual environment model. Based on the Dyna-Q algorithm, we improved the action selection method and added a forward sweep and decision certainty assessment mechanism. The above mechanism is performed inside the robot and depends mainly on the state action value $Q\left(s_{t},a_{t}\right)$ stored in the Q table in the RL algorithm, as shown in the green box on the left of Figure 5.

We define the environmental cognitive task as a Markov Decision Process (MDP), and the standard RL algorithm is a process of interacting with the environment and making mistakes under the MDP framework. This model uses a quintuple $M=\left\{S,A,P,R,\gamma \right\}$. The following formula describes the meaning of each element:

S represents the set of states *s* in the environment $\left(s\in S\right)$. S is defined as follows, where W and H are the width and height of the environment map, respectively:

(3) $$S=\left\{s=\left(x,y\right)\;|\;1\leq x\leq W,\;1\leq y\leq H\right\}$$

A is a set of discrete finite actions $\left(a\in A\right)$ that can be adopted by a robot. The action set is $A=\left\{a_{1},a_{2}\cdots a_{n}\right\}$.

The MBRL algorithm can build a world model in the learning process, which usually includes the state transition function and reward function. In this model, P is the probability of state transition, which represents the probability that the robot is in a certain state s in the process of interacting with the environment and taking action to transfer to state $s_{t+1}$.

R is the reward function of this model, and $R\left(s,a\right)$ is used to measure the instant reward obtained by the robot after taking action a in state s. Specific definitions of R and P are given in the following sections.

In the Q value updated formula adopted by the Dyna-Q algorithm, γ, as a discount factor, is used to discount the maximum expected value in the future.

### 3.2. Brain-Inspired Environmental Cognitive Computing Model

#### 3.2.1. Design of Action Selection Mechanism Based on Forward Prediction and Decision Certainty Assessment

**Figure 6:**
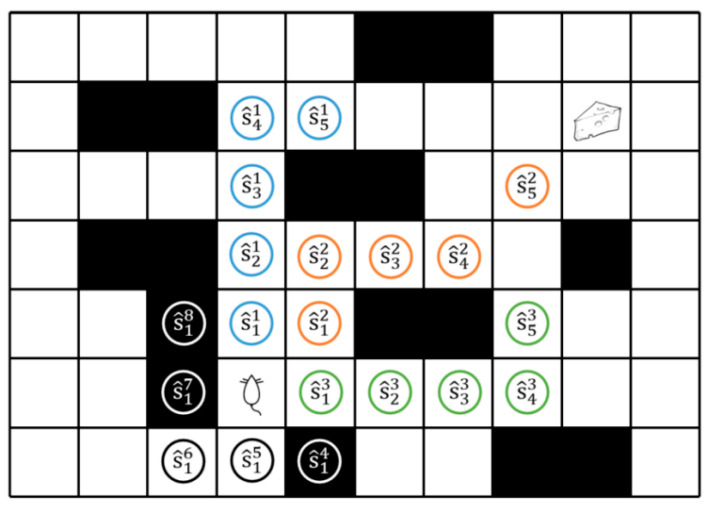
Forward sweep schematic. The sweep is performed on the environment map inside the robot, and the maximum sweep depth is 5. The obstacles shown in Figure 6 have been detected by the robot. The robot sweeps in different directions, as shown in Figure 6, sweeping in directions 1, 2, 3 moving toward the target, represented by blue, orange, and green circles respectively; directions 4, 7, 8 are obstacles, so the sweep stops, represented by white circles; directions 5 and 6 are directions away from the goal, and the accumulated Q value of the sweep is mostly negative, represented by black circles.

This model mainly simulates “mental travel” in an animal’s brain when it is at a difficult decision point to help the robot make better decisions from a long-term perspective. The following is the specific process:

1. Take the current state *s_t_* of the robot as the starting point.
2. In the first sweep step, n actions in the action set A are simulated in sequence in $s_{t}$ to reach n subsequent potential states ${\hat{s}}_{1}^{1}\;\sim \;{\hat{s}}_{1}^{n}$, and the action value $Q\left(s_{t},a_{i}\right)$ of the ith state is accumulated to ${Q\_sweep}_{i}$, respectively. i is the ith direction of the sweep. The range of i is the same as the number of actions in the action set A ,i∈[1, n]. ${\hat{s}}_{j}^{i}$ is the state reached during the sweep, and ${Q\_sweep}_{i}$ is the Q value accumulated in the ith direction during the sweep. Figure 6 shows the forward sweep mechanism in the environment:
3. Then, the ${certainty}_{j}$ of the current depth j is calculated. j is the current sweep depth, dynamically adjusted by the decision certainty in the sweep process, 1≤j≤Max_Depth. If the certainty exceeds the threshold, the sweep stops.
  (4) $$softmax({Q\_sweep}_{i})=\frac{e^{\beta {Q\_sweep}_{i}}}{\sum_{i=1}^{n}e^{\beta {Q\_sweep}_{i}}}$$
  (5) $${certainty}_{j}=max\left(softmax\left({Q\_sweep}_{i}\right)\right)-submax\left(softmax\left({Q\_sweep}_{i}\right)\right)$$
  (6) $$a_{i}=argmax\;{Q\_sweep}_{i}$$

If the decision certainty is greater than the threshold *SweepCertThr*, the sweep stops, and the robot selects the initial action $a_{i}$ taken by the branch with the highest cumulative state action value in n directions.

4. If the certainty does not exceed the threshold, the next sweep will be carried out based on the potential state ${\hat{s}}_{j}^{i}$ reached by the previous sweep. After the softmax function is non-negative and normalized, the value range of ${Q\_sweep}_{i}$ is (0,1), and the value range of the difference between the processed maximum value and the second largest value is also (0,1). Therefore, setting of the threshold SweepCertThr is also a decimal from 0 to 1, and the specific value is fine-tuned by the experimental process.

Unlike the first sweep step, in order to reduce computational complexity, the sweep of the next step will not extend n branches separately but will determine the action $a_{i}$ to be simulated in this step and the possible next state ${\hat{s}}_{j}^{i+1},$ according to the maximum state-action value $Q\left({\hat{s}}_{j}^{i},a_{i}\right)$ in the Q table. At the same time as the sweep, the robot accumulates the state action value $Q\left({\hat{s}}_{j}^{i},a_{i}\right)$ of each direction in each depth sweep to ${Q\_sweep}_{i}$ and uses the discount factor, which decays with the increase in sweep depth, to reduce the weight of the distance. ${discount}_{j}$ is the discount factor, which decreases with the increase in sweep depth.

(7) $${Q\_sweep}_{i}={Q\_sweep}_{i}+{discount}_{j}\cdot Q\left({\hat{s}}_{j}^{i},a_{i}\right)$$

**Figure 7:**
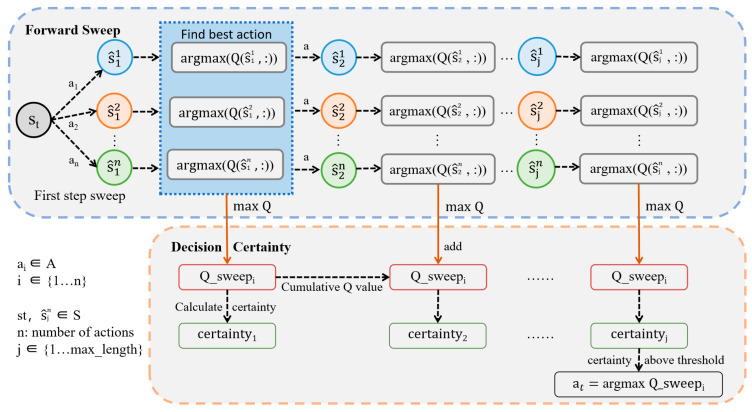
Forward sweep and action selection mechanism. It is performed inside the robot, starting from the current state $s_{t}$, to simulate the new state ${\hat{s}}_{j}^{i}$ after taking various actions and to make a decision on the cumulative Q value of each sweep direction during the sweep process. If the certainty of a certain sweep depth exceeds the threshold, the sweep will end and the sweep direction with the maximum cumulative Q value will be selected as the final action output. The blue oval dashed box represents the forward sweep process, where the blue, orange, and green circles represent sweeps in different directions, and the blue square dashed box is the process of finding the best action. The orange oval dashed box is the process of calculating decision certainty, where different colored oval boxes are used to distinguish different variables.

After the state action values are accumulated in n directions of the current depth, the robot will judge the decision certainty according to the method mentioned in step 3 according to the n accumulated values ${Q\_sweep}_{i}$, until the decision certainty exceeds the threshold, and the robot selects the initial action $a_{i}$ with the maximum cumulative Q value. Figure 7 shows the overall process of the forward sweep and action selection mechanism and Table 1 explains the model parameters.

#### 3.2.2. Improved ε-Greedy Method

The exploration and exploitation dilemma is also a big challenge for RL algorithms. In traditional RL algorithms, such as Q-learning and SARSA, robots adopt the ε-greedy strategy to solve this dilemma, in that they choose the action with the highest Q value most of the time and behave greedily, but sometimes with a small probability ε of randomly selecting actions, which can reduce the probability of falling into the local optimum.

In this paper, the traditional ε-greedy method is improved. In the above-mentioned forward mechanism, if the decision certainty of each depth does not exceed the threshold after the robot sweeps to the maximum depth, the improved method is adopted and the ε-greedy method randomly selects actions with a certain probability. The purpose of this is to reduce the probability of falling into the local optimum and avoid over-reliance on the experience gained, which is similar to the traditional RL algorithm.

Because the sweep in hippocampal cells does not always reflect the direction of the movement of rats [39], this indicates that the sweep reflects the VTE process in the brain rather than the means of collecting external sensory information. Exploratory behavior occurs when mice have very limited experience, while VTE occurs when animals have extensive experience but need to complete specific tasks.

Therefore, the greedy factor $\varepsilon_{vte}$ we used was not set to a fixed value, but in a way that gradually decreased. $\varepsilon_{vte}$ is large in the early stages of training, as robots tend to randomly select actions and explore as many unknown states in the environment as possible. As the number of iterations increases, $\varepsilon_{vte}$ constantly decays to the final degree factor ε, and gradually tends to use the optimal strategy obtained to accelerate the convergence of the robot. In the formula, episode  is the current number of training rounds, and Max_ Episode is the maximum number of training rounds.

(8) $$\varepsilon_{vte}=\frac{3\varepsilon }{1+2\sqrt{\frac{episode}{Max\_Episode}}}$$

The complete pseudo code of the action selection mechanism is as follows:

**Algorithm** **1: Forward Sweep Policy & Improved ε-Greedy Policy****1****INPUT:**     Q, $s_{t}$**2****OUTPUT:** $a_{t}$**3****Initialization:** *n*, *Max_depth*, *SweepCertThr***4***discount =* $\gamma^{\;(1\;:\;max\_depth)}$  // Discount factor, decays with depth.**5**${\hat{s}}_{j}^{i}\leftarrow 0$                             // Initialization of arrived state of each step of sweep**6**Q_sweep ←0             // Initialization of cumulative Q-values after sweeping. **7**certainty←*0*                   // Initialization of decision Certainty of each sweep step. **8**j←1                              // Initialization of current sweep depth**9**
**10****for** i=1 : n **do****11**
**12**   ${\hat{s}}_{1}^{\;i}\leftarrow move(s_{t},a_{i}$*)*   // Predict next state by current state $s_{t}$ and action $a_{i}$
**13**   ${Q\_sweep}_{i}\leftarrow {discount}_{1}\cdot Q(s_{t},a_{i})$
**14****end****15**${certainty}_{1}\leftarrow \;\left|max(softmax(Q\_sweep))-submax(softmax(Q\_sweep))\right|\;$**16****while** j<Max_depth **&** ${certainty}_{j}<$ *SweepCertThr* **do****17**      *j*++**18**      **for** i=1: n **do****19**            $a\;\leftarrow \;argmax(Q({\hat{s}}_{j}^{i},\;:))$      // Find best action in this sweep branch**20**            ${\hat{s}}_{j+1}^{i}\leftarrow \;move({\hat{s}}_{j}^{i},\;a)$              // Take all actions except reversing**21**            ${Q\_sweep}_{j}={Q\_sweep}_{j}+{discount}_{j}\cdot Q({\hat{s}}_{j}^{i},\;a)$
**22**      **end**
**23**      calculate ${certainty}_{j}$
**24****end****25****if** j==Max_depth **&** ${certainty}_{j}<SweepCertThr$ **then****26**      Generate random number *randN*, *randN*
∈(0,1)
**27**         **if** $randN>\varepsilon_{vte}$ **then****28**            $a\leftarrow argmax(Q(s_{t},\;:))$
**29**         **else**
**30**            a←random action **end****31**         **else**
**32**            $a\leftarrow softmax({Q\_sweep}_{i})$
**33****end**

#### 3.2.3. Reward Function R and State Transition Model P

The reward function of this model is as follows, including reward when reaching or approaching the goal and punishment when hitting the wall or leaving the goal. The reward for distance information follows the Gaussian distribution. The closer the robot is to the goal point in the environment, the greater the reward intensity. This is similar to the GPS sensor on a real robot, which can calculate the distance between the current position and the goal. The robot evaluates the reward in the following ways: $r_{hold}$ is the initial value of the reward function, and $r_{near}$ is the coefficient of reward for the robot approaching the goal. The closer to the destination, the greater the overall reward: $r_{neg\;}$ is the reward (punishment) when encountering obstacles, and $r_{goal}$ is the reward for reaching the goal.

(9) $$R=\left\{\begin{array}{c} r_{goal},arrive\;goal\\ {r_{near}\cdot e}^{-\frac{distance}{2\sigma^{2}}},if\;get\;closer\;to\;goal\\ r_{neg},\;Hit\;a\;wall\\ r_{hold},\;else \end{array}\right.$$

In order to better abstract the function of the hippocampus, we used a simple statistical method to model the state transition model P (s′ | s, a) of MBRL. When the environmental states are discrete, each $\left(s_{t+1},s_{t}{,a}_{t}\right)$ can be stored in discrete triples. The robot counts the number of times to reach a specific subsequent state $s_{t+1}$ after taking action $a_{t}$ in the current state $s_{t}$, and takes its proportion to the total number of times to reach all possible subsequent states s′ as the state transition probability $P\left(s_{t+1},s_{t}{,a}_{t}\right)$. The higher the access frequency of the robot, the greater the discount factor when executing the value iteration, and we can realize the internal representation of the environment under the stable environment structure.

(10) $$count\left(s_{t+1},s_{t}{,a}_{t}\right)=count\left(s_{t+1},s_{t}{,a}_{t}\right)+1$$

(11) $$P\left(s_{t+1}\;|\;s_{t}{,a}_{t}\right)=\frac{count(s_{t+1},{\;s}_{t},\;a_{t})}{\sum_{i\in s\prime }count(\;i,{\;s}_{t},\;a_{t})}$$

We also used a model-based value iteration method to update the Q value, and the reward was based on the observed value after taking the current action. The transition between task states is presented in the form of probability. The state transition function $P\left(s_{t+1}\;|\;s_{t}{,a}_{t}\right)\;$ is the probability distribution of all possible states. The influence of the future Q value on the current Q value is measured by the state transition probability. The more times the state is visited during the training process, the more significant is the effect in the model.

(12) $$Q(s_{t}{,a}_{t})=Q(s_{t}{,a}_{t})+\alpha \cdot (R+\gamma \cdot P(s_{t+1}|s_{t}{,a}_{t})\;\cdot \;maxQ(s_{t+1}{,a}_{t+1})-Q(s_{t}{,a}_{t}))$$

In the process of training the robot to find the goal point, consistent with Dyna architecture, we saved the previous and next states and action rewards $\left({s_{t},s}_{t+1}{,a}_{t},R\right)$ for each step. After reaching the goal point in an episode, the robot randomly extracted the saved experience from the model and learned the state value function internally, which is consistent with Dyna-Q’s planning process. Simulation training in the virtual environment model can improve the convergence property of the model.

The pseudo-code of the algorithm is as follows:

**Algorithm 2: Brain-Inspired Dyna-Q Algorithm with VTE mechanism****1****Initialization:** *start*, *goal***2**Q(s,a),count(s’,s,a), P (s’,s,a)←0,∀s ∈ S,  a ∈ A**3****for** episode=1 : Max_Episode **do****4**     Model ←0 , $s_{t}\leftarrow start$
**5**     **while** not arrive *goal*
**&**
step<Step_Max **do****6**          Get action $a_{t}$ by forward sweep policy**7**          Take action $a_{t}$ to get $s_{t+1}$ and reward**8**          $distance\leftarrow |\;s_{t+1}-goal\;|$
**9**          **if** $\;R\neq r_{neg}$ **then**
**10**          $count(s_{t+1},s_{t},a_{t})=count(s_{t+1},s_{t},a_{t})+1$
**11**          $P\left(s_{t+1}\;|\;s_{t}{,a}_{t}\right)=\frac{count(s_{t+1},{\;s}_{t}\;,\;a_{t})}{\sum_{i\in s\prime }count(\;i,{\;s}_{t},\;a_{t})}$
**12**          **else** $count(s_{t+1},s_{t},a_{t})\;,P\left(s_{t+1}\;|\;s_{t}{,a}_{t}\right)\leftarrow 0$**13**          **end**
**14**$Q(s_{t},a_{t})\leftarrow Q(s_{t},a_{t})+\alpha \cdot (R+\gamma \cdot P\left(s_{t+1}\;|\;s_{t}{,a}_{t}\right)\cdot maxQ(s_{t+1},a_{t+1})-Q(s_{t},a_{t}))$**15**          $Model(step)\leftarrow s_{t},s_{t+1}{,a}_{t},R$
**16**          step++
**17**     **end**
**18**     if step<=step_old **then****19**          Model_save ← Model // Update model**20**     **end**
**21**     **for** i=1 : n **do**     // Model learning inside of robot**22**          $s_{t}\leftarrow$ random experienced state in Model**23**          $a_{t}\leftarrow$ random action taken at state $s_{t}$ in Model**24**          Take action $a_{t}$ to get $s_{t+1}$
**25**          R ← reward at state $s_{t+1}$ in Model**26**          $Q(s_{t},a_{t})\leftarrow Q(s_{t},a_{t})+\alpha \cdot (R+P\left(s_{t+1}\;|\;s_{t}{,a}_{t}\right)\cdot maxQ(s_{t+1},a_{t+1})-Q(s_{t},a_{t}))$
**27**     **end**
**28****end**

## 4. Results

In this section, we have conducted several sets of experiments to test the performance of our model. In this paper, a simulation experiment was carried out in the T-choice maze, and the navigation performance of the forward prediction mechanism and the navigation performance without it were compared. The advantages of this mechanism were preliminarily demonstrated and the characteristics of this model similar to the rat VTE mechanism were verified. Then, this paper conducted a complex environment navigation experiment and tested the environmental cognition and path planning ability of this model in the complex maze environment with static and dynamic obstacles. We compared SARSA, the Dyna-Q algorithm, and this algorithm without the decision certainty assessment to verify the advantages of this algorithm.

### 4.1. Environment and Parameter Configuration

For convenience, the experimental environment in this paper is set up as a number of square grids, each of which is the same size and represents a specific state s in space. The white grid represents the accessible road, and the black grid represents the obstacle. Our robot was set up as a four-wheel omni-directional mobile robot. The robot was equipped with a laser sensor that could sense the presence of surrounding obstacles.

**Figure 8:**
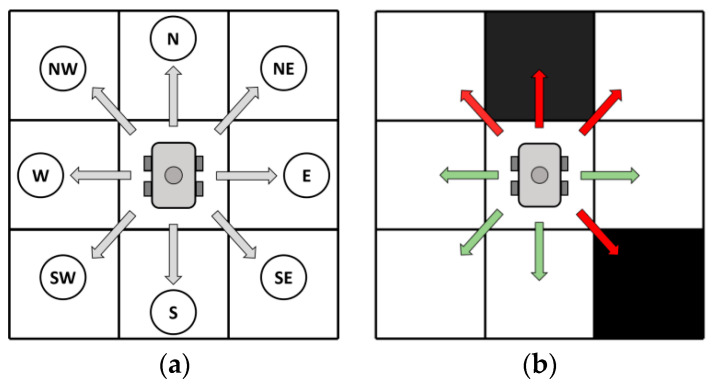
Robot action space. (**a**) The eight actions of the robot; (**b**) The green arrow indicates the actions that the robot can take after encountering obstacles, and the red arrow indicates the actions that cannot be taken.

Robot action set A={a1,a2,a3,a4,a5,a6,a7,a8} included eight action primitives: move one unit step in eight directions, namely, north, northeast, east, southeast, south, southwest, west, and northwest, and enter the adjacent state. When there are obstacles in the environment, the robot’s movement rules are shown in Figure 8. The green arrow indicates the actions that the robot can take, while the red arrow indicates the actions that the robot cannot choose. It is particularly important to note that when the robot is adjacent to the obstacle, it cannot use the oblique action that may collide with the obstacle. The robot moves at a constant speed and moves one grid per unit time step.

Rats will use a variety of sensory information when exploring the environment. Some brain-inspired models [40] introduce visual and olfactory perception modules for the robot and use scene and odor information to guide the robot. This model simulated the characteristics of rats in the maze and other experimental scenes. It was assumed that the robot has a GPS sensor, which can sense its position to obtain its state, and then the Euclidean distance was obtained from the goal, which can simplify the calculation characteristics of the model and make the model more biologically reasonable.

We used MATLAB R2021a software to carry out simulation experiments on the computer. The parameter configuration of this model and the simulation experiments is shown in Table 2, including the learning rate α, greedy factor ε, Max_Step, reward R, etc., in the experiment. In order to ensure the fairness of the experiment, all methods were compared in the same parameter settings.

### 4.2. Experiment on Spatial Cognition Experiment in T-Maze: Demonstrates the Bionic Characteristics of This Model

**Figure 9:**
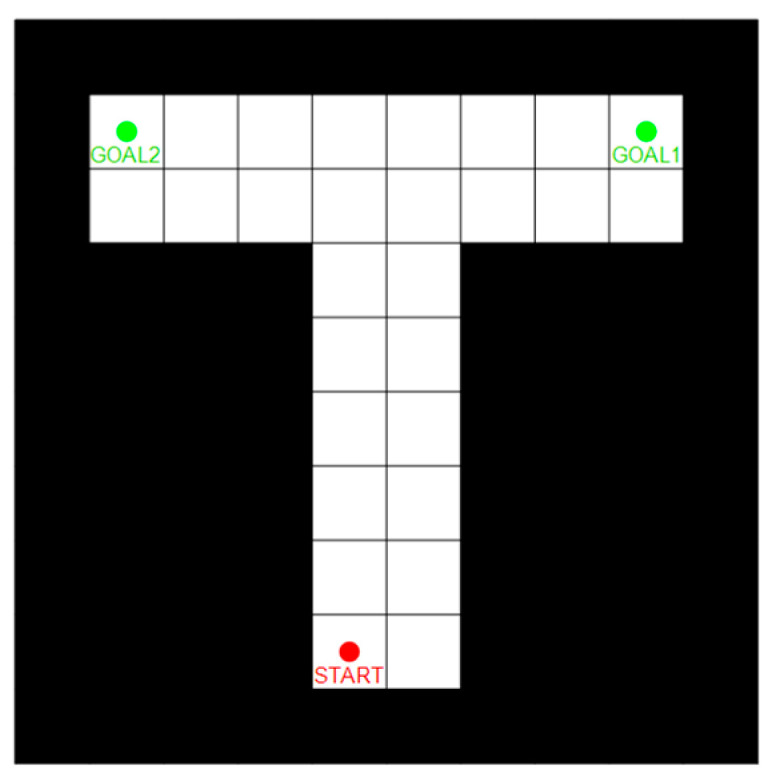
T-maze. The red circle is the starting point and the two green circles are the goal points, which are applied to different experimental conditions, respectively.

Some scholars [41,42] have tested the VTE mechanism of rats in the T-choice maze. They verified that the rat VTE process usually occurs at the high-cost selection point and gradually disappears as action selection tends to stabilize, but when reward transmission changes accidentally, VTE will reappear. As shown in Figure 9, this paper simulated a small T-maze, and tried to verify whether the model had the above characteristics, and then verified the advantages of the forward sweep mechanism for action selection through comparison.

In the T-maze, the red circle is the starting point of the robot and the two green circles are the destination. The rats start from the red point and explore freely to find the destination. When they reach the destination or exceed the maximum step length, the experiment ends.

#### 4.2.1. Comparison between Using the Forward Sweep Mechanism and Non-Sweep

First, we tested the forward sweep mechanism. The robot navigated the goal point 1, and we compared the navigation performance of the robot when using forward sweep and non-sweep (set sweep depth to 0).

**Figure 10:**
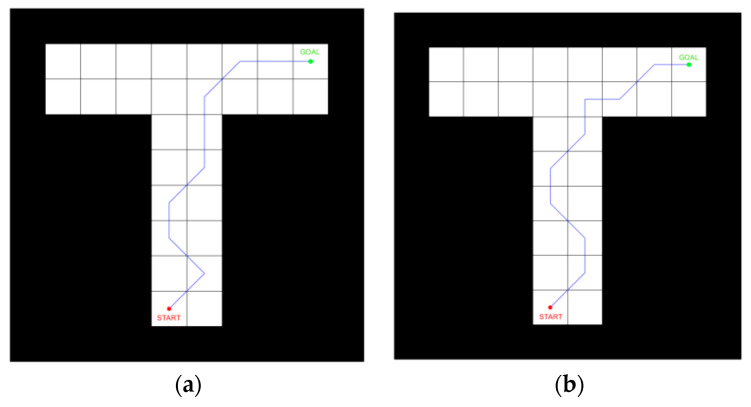
Results of the T-maze experiment. (**a**) The route planned by forward sweep; (**b**) The route planned by the non-sweep method. The red circles in the figure represent the starting point, while the green circles represent the ending point, and the blue lines represent the navigation path.

Our model successfully simulated the goal-oriented behavior of rats in the maze. Through training and reward, the robot could successfully reach a specific destination, proving that this model had the same spatial cognitive ability as animals. Figure 10 is the path planning result of the T-maze experiment.

**Figure 11:**
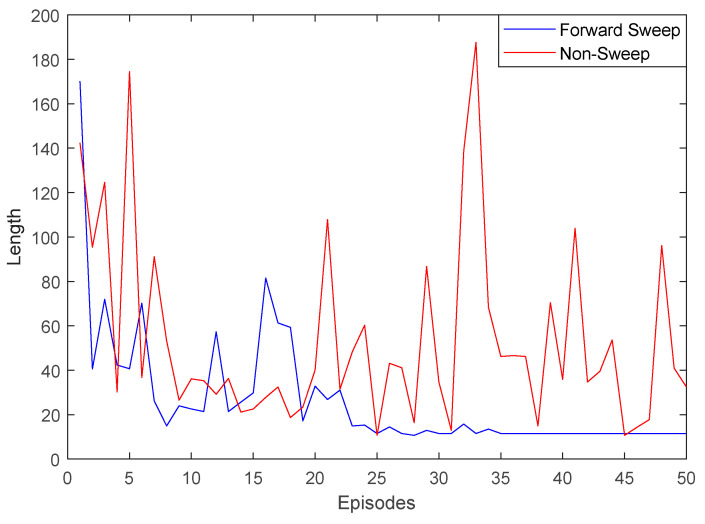
Learning curve of forward sweep and non-sweep.

Because the maze structure was relatively simple, there was no large gap in the planned path length, but there was a significant gap in its convergence property. After 20 episodes of training, the path length of this model was significantly reduced, while the path length without the forward sweep method was still divergent. As can be seen from Figure 11 below, the forward sweep mechanism could improve the convergence property of robot learning and thus improve navigation efficiency.

#### 4.2.2. Characteristics Similar to Rat VTE Mechanism

**Figure 12:**
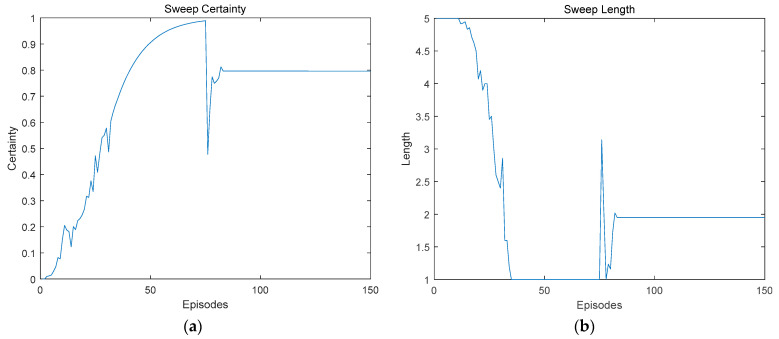
A similar phenomenon to rat VTE was observed in this model. (**a**) Decision certainty; (**b**) Forward sweep length.

In this group of experiments, the robot used the model in this paper, taking goal point 1 as the destination in the first half of the training, and switching the end point to goal point 2 in the second half of the training. Figure 12 shows the length of the forward sweep of the robot and the decision certainty during the training process. It can be seen from Figure 12 that, in the first half of the training process, the certainty was constantly improved, the sweep length was continuously reduced, and the action selection of the robot tended to be stable. When the goal point changed, VTE occurred again, the decision certainty decreased, and the sweep length increased.

**Figure 13:**
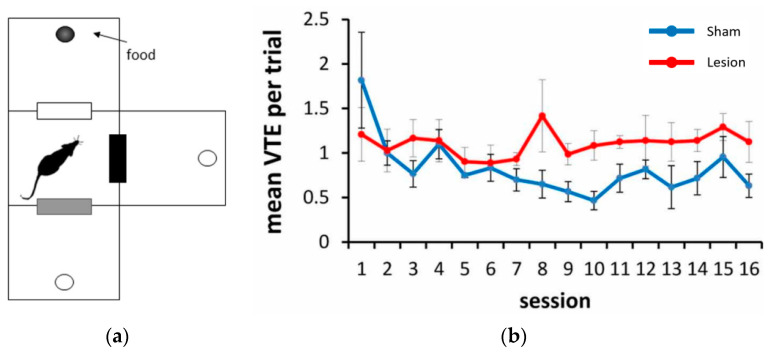
Phenomenon of rat VTE mechanism observed in neurophysiology [43]. (**a**) Rats are trained to find food rewards in the three-choice maze, and the black circle is the container for placing food; (**b**) Average VTE in the three-choice test in the 16 episodes.

The above phenomenon was basically consistent with the characteristics of the rat VTE mechanism found by researchers. Bett et al. [43] trained rats to seek food rewards in a three-choice maze with a fork in the road and tested them under sham surgery (sham) or hippocampus lesion surgery (lesion) conditions of the rat hippocampus. The results showed in Figure 13 that, as training progressed, VTE activity in rats with a normal hippocampus gradually decreased, while VTE activity in rats with a damaged hippocampus actually maintained a high level. This physiological experiment proved that, in the spatial memory task, the damaged hippocampus showed similar levels of VTE before and after recognizing the reward position. By contrast, rats with sham injuries showed higher VTE behavior in the experiment before finding the reward position rather than in the experiment after finding it.

The model also showed similar phenomena with neurophysiology, and the above experiments preliminarily confirmed the biological rationality of the model. As rats gained more experience in the environment, the frequency of VTE gradually decreased, and the hippocampus sweep also moved in the final direction. After the decision certainty exceeded the threshold, VTE no longer occurred, the rats’ moving path became stable, and place cells in the hippocampus no longer generated a forward firing sequence, which meant that the frequency and depth of forward sweep within the robot was reduced.

### 4.3. Path Planning in Complex Maze Conditions: Testing the Navigation Capability of Our Model

We tested the model in an environment with static and dynamic obstacles. The main purpose of the experiment was to allow the robot to explore and find the best path to the destination without prior knowledge of the environment, while avoiding all obstacles.

#### 4.3.1. Static Obstacle Environment

**Figure 14:**
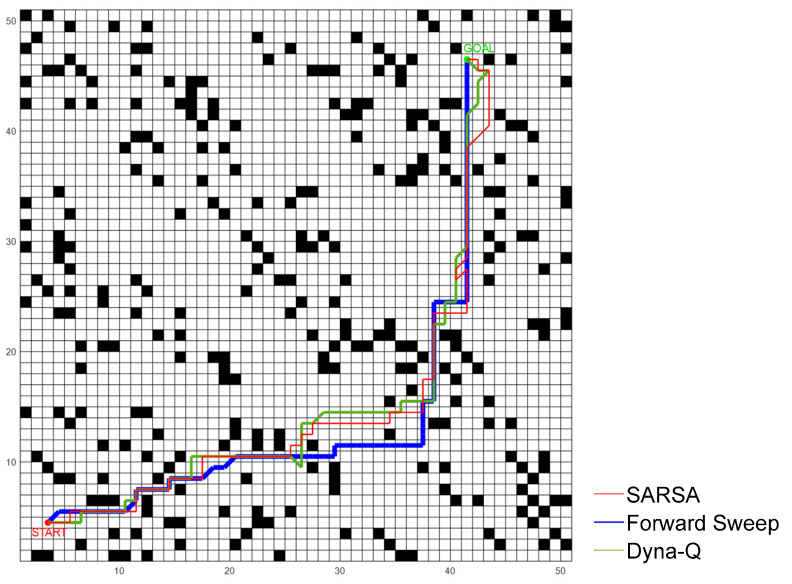
The paths planned by each method in the static environment.

First, we conducted experiments in an environment with static obstacles. The following Figure 14 shows the path the robot planned after 1500 episodes of training. Each algorithm was repeated 10 times and the average value was calculated. By comparison, it can be found that the path planned by our model was more straight, while the path planned by SARSA and Dyna-Q had many twists and turns, and sometimes would also choose the direction of deviation.

**Figure 15:**
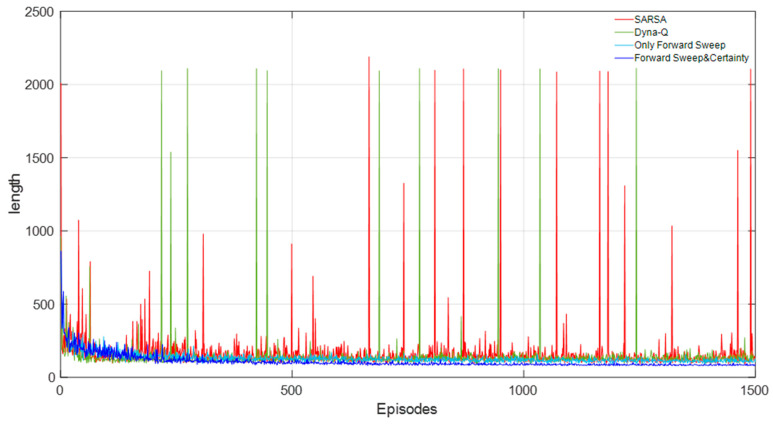
Comparison of learning curves of four methods in a static environment.

Figure 15 shows the learning curves of the four methods. It can be seen that the convergence property of our model was better and the average path length after convergence was lower, while the other three methods still fluctuated and tended to diverge from time to time.

According to statistics, the average path length of our model was 78.26 units, while SARSA and Dyna-Q both exceeded 88 units, which showed that this model had significantly improved the learning speed and environmental cognitive efficiency of the robot. Table 3 shows the length of the robot’s path.

**Figure 16:**
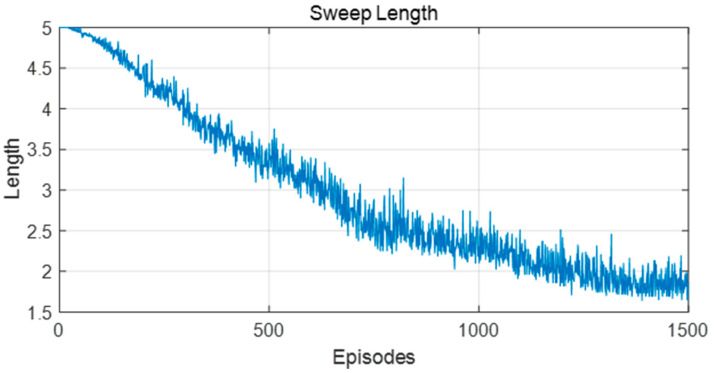
Changes in the forward sweep length during the learning process in the static environment.

**Figure 17:**
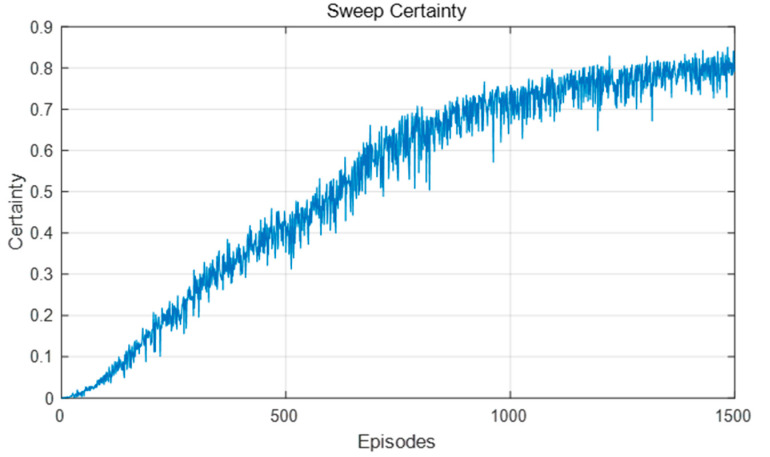
Changes in the forward sweep decision certainty during the learning process in the static environment.

Figure 16 and Figure 17 show the changes in forward sweep length and decision certainty in the training process of this model. It can be seen that with the continuous improvement of the decision certainty of the robot, the action selection tended to be stable and the sweep depth also gradually decreased.

#### 4.3.2. Dynamic Obstacle Environment

**Figure 18:**
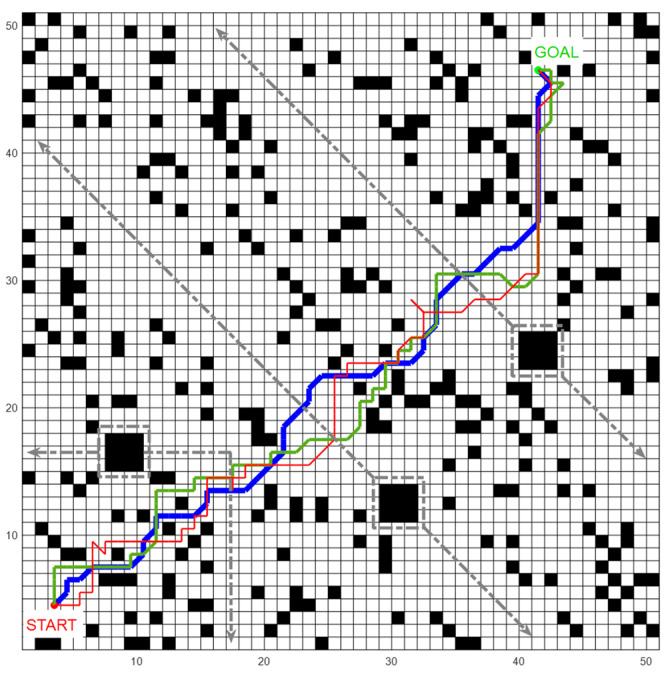
Path planned by each method in a dynamic environment. The red, blue, and green lines in the graph represent the paths planned by SARSA, our algorithm, and Dyna-Q, and the dashed gray lines represent the motion trajectories of three obstacles.

Next, we added three dynamic obstacles to the environment, all of which were 3 × 3 black grids in size. Each dynamic obstacle moved back and forth along the track, as shown by the gray line in Figure 18, and the movement speed was constant at half the robot’s speed, i.e., the obstacles moved half a grid per unit step.

Figure 18 shows the path planned by the three methods after 1500 episodes. It can be seen that the robot could effectively realize environmental cognition and path planning in a dynamic environment and successfully avoided obstacles. It can be seen that the path planned by our model was more gentle, while SARSA and Dyna-Q led to detours, resulting in redundant paths.

**Figure 19:**
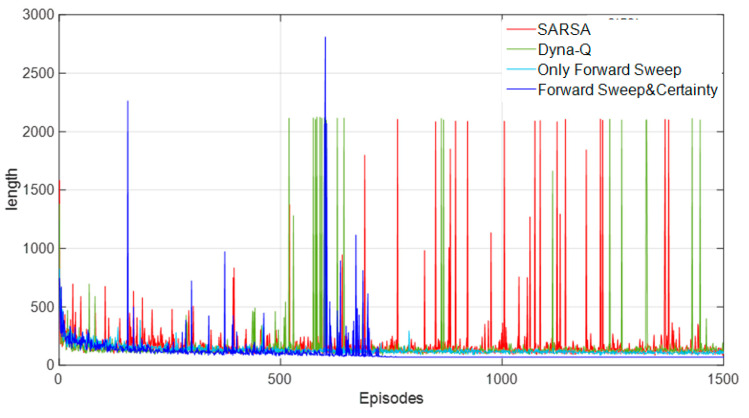
Comparison of learning curves of four methods in a dynamic environment.

In terms of learning efficiency in Figure 19, we can see that the speed of convergence of this model still had obvious advantages over the other two methods. The average path length of our model was shorter, the convergence property was better, and the region was stable at the later stage of training, while the other three methods still had difficulty in converging at the later stage, with large fluctuations. Table 4 shows the length of the robot’s path.

**Figure 20:**
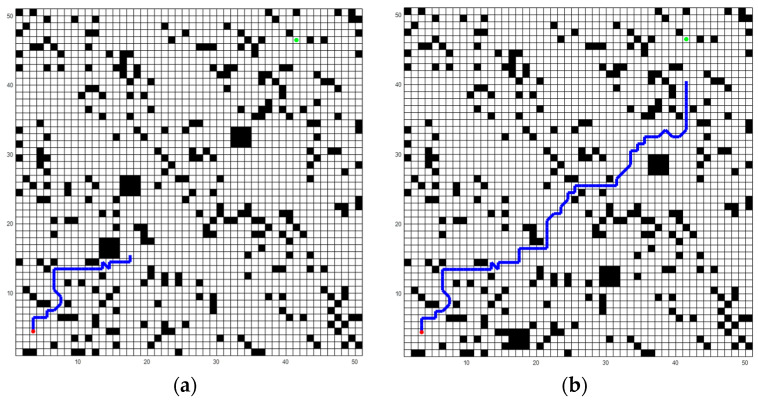
The trajectory of the robot in a test using this model, represented by blue lines. (**a**) The robot moves 30 steps; (**b**) The robot moves 74 steps. The black squares in the figure represent obstacles.

Figure 20 shows the later stage of robot training. It can be seen that with the progress of training, the robot could successfully bypass dynamic obstacles and move toward the destination.

**Figure 21:**
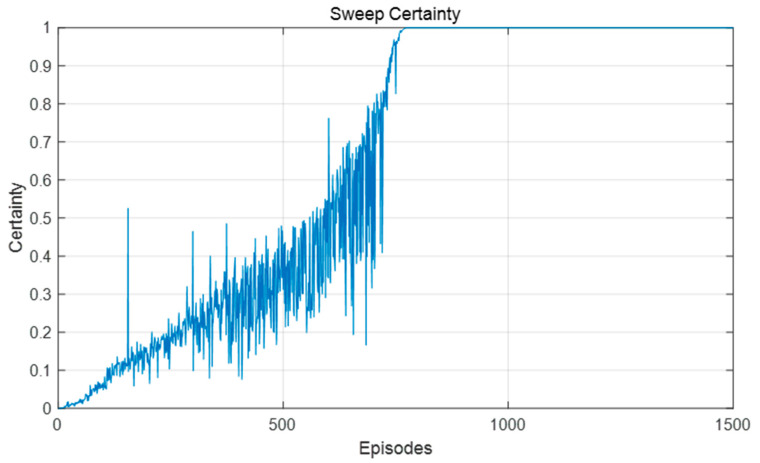
Changes in the forward sweep decision certainty in the dynamic environment.

**Figure 22:**
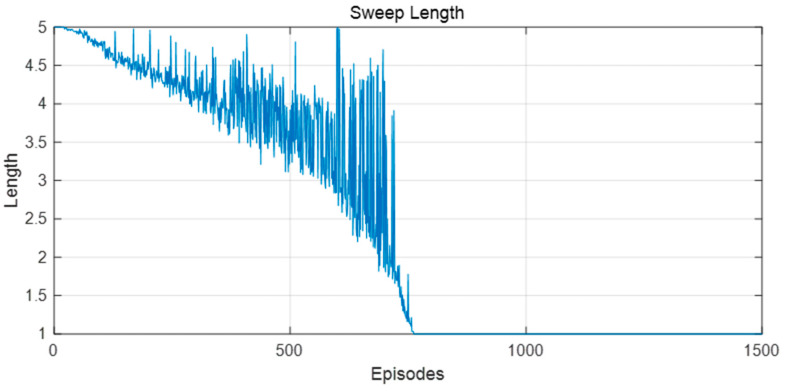
Changes in the forward sweep length in the dynamic environment.

Because the dynamic obstacles moved to different positions in each training episode, the convergence speed of the algorithm was slower than that in the static environment. Whenever the robot encountered a dynamic obstacle, it may choose different actions at the same position than when it encountered a dynamic obstacle. Accordingly, due to the increasing difficulty of spatial cognition and path planning, the frequency of VTE was also higher, the length of forward sweep was longer, and the decision certainty also fluctuated greatly, as shown in Figure 21 and Figure 22. In short, the sweep depth was reduced and the decision certainty was improved by this algorithm, indicating that this algorithm can be adapted to the dynamic environment.

## 5. Discussion

The experimental results showed that this model can improve the efficiency of action selection and learning, improve robot performance in environmental cognitive tasks, and is superior to the traditional model-free and model-based RL algorithms.

### 5.1. The Significance of the VTE-Inspired Mechanism in Our Algorithm

In the T-maze experiment, this model reproduced key features of the VTE mechanism in rats and other mammals, which was consistent with experimental results from physiological research. At the beginning of maze training, rat hippocampal activity is strong, and the VTE process is more obvious. Rats frequently simulate the possibility of future paths in the brain. With an in-depth understanding and familiarity with the environment, the role of VTE is weakened and the choice of action tends to be fixed. Hippocampus forward sweep and decision certainty assessment, corresponding to the action selection model, have a strong effect when the decision was uncertain at the initial stage of the robot’s exploration. The sweep length was longer and the robot performed a simulated behavior similar to that of rats, searching for possible future paths to improve the navigation effect. With continuous training, the decision certainty was gradually enhanced, the role of VTE was constantly weakened, the sweep length of this model was also gradually reduced, and the selection of robot actions tended to be stable. The above experimental phenomena proved the biological rationality and advantages of introducing the forward prediction mechanism and decision certainty assessment into traditional RL algorithms.

Current research to improve the Dyna-Q algorithm is rarely performed from the perspective of brain-like computing. For example, Pei et al. [14] improved Dyna-Q by incorporating heuristic search strategies, a simulated annealing mechanism, and a reactive navigation principle into the algorithm. Some neurophysiological studies, such as Bett’s work [43], have focused on verifying the characteristics of the VTE phenomenon in rats and the activities of brain areas such as the hippocampus through biological experiments. Some brain-inspired models, such as those by Khamassi [35] and Stoianov [37], also focused on mathematical modeling to reproduce and explain the possible causes of rat physiological phenomena. However, they did not apply it further to RL and robot path planning. In this paper, we combine the neural mechanism of rats with the RL method, not only verifying the rationality of current biological research on VTE by reproducing it on agents but also providing a possible application in robot navigation with the VTE mechanism.

### 5.2. Improved RL Algorithm to Achieve better Performance in Path Planning Tasks

Traditional MFRL frameworks, such as SARSA, learn through rewards obtained through direct interaction with the external environment, but this will result in high computational costs and slow convergence. From our experimental results, we can see that SARSA’s performance in navigation tasks was significantly poorer than that of the two other model-based algorithms. Navigation by SARSA was inefficient and led to many meaningless explorations in the environment. When SARSA was applied in a large maze, meaning that the rewards were more sparse, not only its training time but also the path length apparently increased. In addition, any environmental changes in the dynamic maze caused SARSA to diverge again, which was fully illustrated by the experimental results.

Dyna-Q, as a model-based algorithm, stores the experience obtained from direct interaction with the environment in the robot’s environment model based on the model-free learning framework and accelerates the learning speed and improves the accuracy of decision making by learning the model. This can effectively reduce the number of interactions with the environment and improve navigation efficiency. The experimental results showed that Dyna-Q’s convergence speed and frequency of post-convergence fluctuations were better than those of the model-free SARSA algorithm. However, even Dyna-Q still performed poorly in dynamic obstacle environments. The dilemma of balancing exploration and exploitation makes both SARSA and Dyna-Q prone to local optimization, resulting in long planned paths and poor navigation performance, especially in complex environments.

By contrast, the algorithm we present alternately attempted potential paths virtually and dynamically adjusted the sweep length according to the decision certainty, which allowed the robot to fully consider the possible state in the future and the effect of each action when making decisions, helping to comprehensively evaluate all action options. At the same time, the forward sweep mechanism further reduced the number of interactions with the real environment, making full use of the experience gained and reducing the cost of the navigation process.

In addition to the forward sweep mechanism, decision certainty assessment also plays an important role in our algorithm based on the experimental results. In our algorithm, the certainty of forward sweep increased with training, and contextual preference for specific goal positions was formed through learning, which was similar to spatial cognition of animals. Accordingly, after having enough confidence in the decision, the sweep length also decreased. This dynamic mechanism allowed the robot to fully explore the environment and better learn action selection. And the improved ε-greedy method, similar to the animal VTE mechanism, enabled the robot to explore the environment in the early stage of training and to use the existing experience in the later stage, which effectively resolved the exploration and exploitation dilemma.

## 6. Conclusions

This paper proposes an improved Dyna-Q algorithm inspired by the VTE behavior of rats and applies it to robot navigation. By imitating the forward sweep mechanism in the hippocampus and decision certainty measurement in the striatum, the algorithm can make robots navigate autonomously, effectively speed up convergence and learning, and solve the dilemma of balancing exploration and exploitation compared with the SARSA and Dyna-Q algorithms.

We carried out a series of simulated experiments to verify the validation of the proposed algorithm. In the T-maze experiment, the algorithm made the agent behave similarly to the VTE behavior of rats, which proved the biological plausibility of our algorithm. In addition, we tested the navigation performance of our algorithm in static and dynamic environments and compared it with other RL algorithms, including SARSA and Dyna-Q. The experimental results showed that our algorithm outperformed comparisons in both learning rate and path length. In short, our work can not only provide more evidence for neurophysiological research by reproducing biological findings in robots but also help to improve the Dyna-Q algorithm and be applied to path planning.

However, there is still room for further improvement in our work. Firstly, all experiments in the paper were simulated, which may weaken the practical value of the proposed algorithm and limit the research for some topics, such as improving energy efficiency during navigation, etc. In addition, the calculation of forward sweep depth and decision certainty affects the speed of action selection. All of these shortcomings will be the direction of the next optimization.

## Figures and Tables

**Table 1:** Model parameter description.

Parameter	Meaning
$s_{t}$	Current robot state
${\hat{s}}_{j}^{i}$	Potential states reached during sweep
i	The *i*_th_ direction of sweep direction, i∈[1,n]
j	Current sweep depth, 1≤j≤Max_depth.
${Q\_sweep}_{i}$	Q value accumulated in the *i*_th_ direction during sweep
n	The number of actions that can be selected by the robot
${discount}_{j}$	The discount factor decreases with the increase in sweep depth.
SweepCertThr	Decision certainty threshold

**Table 2:** Parameter Configuration of Simulations.

Parameter	Meaning	Value
α	Learning rate	0.1
β	Softmax factor	0.5
γ	Discount factor	0.95
ε	Greedy factor	0.2
σ	Standard deviation	0.35
*SweepCertThr*	Decision certainty threshold	0.5
*Max_depth*	Maximum sweeping depth	5
*Max_Step*	Max steps	2000
*Max_Episode*	Max episodes	1500
*N_Dyna*	Planning steps	50
*n*	Number of actions	8
$r_{hold}$	Initial reward	−0.01
$r_{g}$	Reward for reaching the goal	1
$r_{near}$	Reward for approaching the goal	0.1
$r_{neg}$	Punishment for hitting the wall	−0.02

**Table 3:** Average learning results in a static obstacle environment.

		SARSA	Dyna-Q	Improved Dyna-Q (None-Certainty)	Improved Dyna-Q
Path Length (unit)	min	86.14	86.90	86.07	**76.14**
	max	90.9	91.31	90.14	**80.14**
	mean	88.59	89.70	88.15	**78.26**

**Table 4:** Average learning results in a dynamic obstacle environment.

		SARSA	Dyna-Q	Improved Dyna-Q (None-Certainty)	Improved Dyna-Q
Path Length (unit)	min	81.90	82.73	82.97	**69.11**
	max	94.97	93.80	88.46	**78.49**
	mean	87.66	88.62	85.06	**75.59**

## Data Availability

The data are contained within the article.
